# Plant–Soil Relationships Diminish Under Major Versus Moderate Climate Change in Subalpine Grasslands

**DOI:** 10.1002/ece3.72578

**Published:** 2025-12-11

**Authors:** Tyson J. Terry, Peter Wilfahrt, Diana R. Andrade‐Linares, Khatab Abdalla, Bernd J. Berauer, Michael Dannenmann, Noelia Garcia‐Franco, Jincheng Han, Andreas von Hessberg, Elisabeth Ramm, Ralf Kiese, Ingrid Kögel‐Knabner, Yujie Niu, Michael Schloter, Stefanie Schulz, Martin Wiesmeier, Anke Jentsch

**Affiliations:** ^1^ Disturbance Ecology and Vegetation Dynamics, Bayreuth Center of Ecology and Environmental Research Bayreuth University Bayreuth Germany; ^2^ School of Life Sciences Arizona State University Tempe Arizona USA; ^3^ Ecology, Evolution, and Behavior University of Minnesota St. Paul Minnesota USA; ^4^ Environmental Microbiology Research Group‐ EMRG, Biological Sciences Department University of Limerick Limerick Ireland; ^5^ Agroecology, Bayreuth Center of Ecology and Environmental Research Bayreuth University Bayreuth Germany; ^6^ Plant Ecology Institute of Landscape and Plant Ecology University of Hohenheim Stuttgart Germany; ^7^ Karlsruhe Institute of Technology Institute for Meteorology and Climate Research, Atmospheric Environmental Research (IMK‐IFU) Garmisch‐Partenkirchen Germany; ^8^ Soil Science, School of Life Sciences Technical University of Munich Freising Germany; ^9^ Field‐Crop Systems and Plant Nutrition Nyon Switzerland; ^10^ Institute for Advanced Study Technical University of Munich Garching Germany; ^11^ Bavarian State Research Center for Agriculture Institute for Agroecology and Organic Farming Freising Germany

**Keywords:** experiment, microbial community, nitrogen, plant–soil feedback, plant–soil interaction, productivity, soil warming, subalpine grassland

## Abstract

Plant communities and soil microbial communities influence each other directly and indirectly via the resource pools they modify. Despite apparent sensitivities of plants and microbes to climate, little is known concerning how climate change will affect plant–soil relationships. We conducted a downslope translocation of intact soil–plant mesocosms in subalpine grasslands to mid‐ and low‐elevation sites to determine how climate change (warmer and drier conditions) influences plant–soil relationships. While soil nutrient pools and microbial composition were key determinants of plant community characteristics under control and moderate climate change (+1°C, +8 days growing season), these relationships diminished under major climate change (+3°C, +21 days growing season). Positive correlations of fungi and nitrogen‐fixing bacteria for plant growth emerged under moderate climate change and diminished under major climate change. Our findings indicate that climate change effects do not solely impact plant community metrics, soil nutrient pools, and soil microbial community composition, but also a breakdown in the ecological coupling among them. We found evidence of threshold‐like behavior for plant–soil relationships in response to major versus moderate environmental change and that plant community metrics and soil microbial dynamics may become more independent in subalpine grasslands following environmental shifts that accompany climate change.

## Graphical abstract

We quantified differences in plant–soil realtionships following downslope transplants of intact plant soil cores. We found that plant–soil realtionships were maintained following moderate climate change and diminished following major climate change.

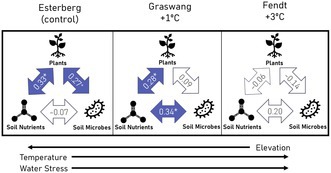

## 1 Introduction

Plant community type and productivity are closely linked to soil resources, the availability of which is mediated by microbial communities that influence both the quantity and uptake by plants (Bardgett and van der Putten 2014; Pugnaire et al. 2019; Elrys et al. 2021). Current trends of increasing global air and soil temperatures (Soong et al. 2020) impact processes of respiration (Atkin and Tjoelker 2003; Frey et al. 2013; Wang et al. 2014), resource availability (Dai et al. 2020), and enzymatic activity (Hammerl et al. 2019) in both plants and microbes (Lu et al. 2013). Unilateral sensitivity of plants or microbes to changes in climate could lead to top‐down or bottom‐up impacts on plants, soil microbes, and soil nutrient pools (Bardgett et al. 2013; Bardgett and van der Putten 2014). While numerous studies have examined the effects of heat and drought on plant and microbial communities independently (Epstein et al. 1997; Menzel et al. 2006; Beierkuhnlein et al. 2011; Leonelli et al. 2011; Wang et al. 2016; Alvarez et al. 2018), we still have a limited understanding concerning how climate change with multiple climate factors will alter the interactions between plants, soil microbes, and soil nutrient pools. To predict the future of natural ecosystems under current climate trends, it is essential to broaden our understanding of how these interrelated processes may shift (van der Putten et al. 2016). Without this knowledge, we cannot reliably determine whether our current understanding of plant responses to changing environments might overlook critical climate‐driven alterations in plant–soil relationships that maintain ecosystem identity and function.

Plant adaptability to climate change will largely depend on their ability to meet resource demands under novel environmental conditions (Ahanger et al. 2016; Tang et al. 2024) despite anticipated changes in nutrient pools and fluxes. In grasslands, plant growth is tightly linked to the ecological stoichiometry of key elements, particularly the ratios of carbon (C), nitrogen (N), and phosphorus (P) (Liao et al. 2023). Under changing climates, nutrient availability is thought to drive patterns of plant growth and resilience (Yu et al. 2015). For example, increased availability of N and P has been shown to enhance the drought tolerance of plant communities (Tang et al. 2024). However, warmer and/or drier conditions that enhance plant nutrient demand may simultaneously alter nutrient abundance, potentially alleviating or exacerbating resource limitations. Warming can accelerate biogeochemical processes such as mineralization, nitrification, and denitrification, thereby influencing N availability and the pools of inorganic forms of P, sulfur (S), and potassium (Dai et al. 2020; Abdalla et al. 2024; Hu et al. 2024). This raises a critical question: will changes in microbial communities increase plant‐available nutrient pools to meet the amplified demands of plants under warmer conditions?

Climate change can alter both the strength and direction of plant–soil microbe relationships (van der Putten et al. 2016; Pugnaire et al. 2019). These relationships can be direct, such as through pathogenic relationships or symbiotic resource acquisition, or indirect via soil microbial processes that regulate resource pools for plants (Bardgett and van der Putten 2014). Shifts in climate can influence the productivity and composition of both plant communities (Wang et al. 2019; Schuchardt et al. 2023) and soil microbial communities (Kaisermann et al. 2017; Gao and Yan 2019; Xu et al. 2023), potentially affecting the abundance and activity of symbionts, pathogens, and competitors (Classen et al. 2015). For example, warmer temperatures are thought to increase abundance but decrease the activity of arbuscular mycorrhizal fungi (AMF) and decomposers that can enhance plant growth and resilience (Pugnaire et al. 2019). Conversely, warming may also promote the abundance of pathogenic bacteria and fungi (Mohan et al. 2014; Rasmussen et al. 2020). In addition to changes in microbial community composition and activity, climate shifts may also modify the strength of plant–soil relationships through changes in plant resource needs (Gargallo‐Garriga et al. 2015; Urbina et al. 2017) and physiological constraints (Atkin and Tjoelker 2003). For example, drier conditions are thought to strengthen associations between plants and AMF (Pugnaire et al. 2019). However, these conditions may also heighten plant susceptibility to pathogens by reducing immunity‐triggering processes (Velásquez et al. 2018). If climatic changes surpass the optimal functional range for these interactions, the benefits of symbionts may diminish, and pathogen pressure may decline as microbes face thermal stress or are replaced by less efficient or less impactful microbial communities (Bradford 2013). Understanding how these dynamics shift under novel climates is critical for predicting the resilience of ecosystems to environmental change.

Our current understanding of plant–soil interactions remains limited, as it is primarily based on studies monitoring the growth of monocultures in growth chambers or green houses, which may not fully capture the dynamics of heterogeneous plant and soil microbial communities (Meisner et al. 2013; Abdalla et al. 2024). To accurately predict ecosystem responses and thus understand natural systems, we require a community‐level perspective that accounts for shifts in plant composition and soil microbial functional groups likely driven by differing sensitivities to climate and shifts in competitive dynamics among distinct groups or taxa following climatic change. Moreover, many studies investigating climate change impacts use a single treatment level, overlooking the potential for threshold‐like or nonlinear responses in both plant and microbial processes to environmental change (Alvarez et al. 2018). Addressing these gaps is critical for understanding the complex interactions shaping ecosystem function and identity under climate change.

Our objective was to investigate how linkages between plants, soil nutrients, and biotic soil conditions change with shifts in abiotic conditions. To address this, we conducted a downslope translocation of plant–soil mesocosms from a subalpine grassland, a system experiencing rapid climatic shifts including warming and increasing drought (Gobiet et al. 2014), that is also characterized by strong plant–soil linkages (Ba et al. 2012; Andrade‐Linares et al. 2023). This approach allowed us to monitor plant–soil relationships under both moderate and major climatic changes that co‐occur with translocations from a high origin site to recipient sites at mid and low elevation, respectively. We hypothesized that (1) the strength of plant–microbe–soil nutrient relationships will increase under both moderate and major environmental changes, as plants increasingly rely on microbial‐mediated nutrient cycling to meet resource demands under altered abiotic conditions, and (2) plant‐microbe and plant‐nutrient relationships will become increasingly critical for maintaining plant productivity and species richness in response to environmental change.

## 2 Material and Methods

We utilized a downslope translocation experiment to expose mesocosms consisting of intact plant–soil from one upslope site to two lower sites with warmer annual air temperatures (+1°C and +3°C, (Zistl‐Schlingmann et al. 2020)) and periods of drier soils (Berauer et al. 2021). We monitored the shift in metrics related to plant–soil relationships following downslope translocation and within a control group that was translocated within the site of origin. To address our first hypothesis, we used matrices of multivariate plant and soil microbial community metrics from each mesocosm across downslope translocations to understand if the strength in the relationship between plants, soil nutrient pools, and soil microbial groups changes between translocation sites. To address our second hypothesis, we used correlation values of individual soil and microbial metrics with plant biomass and plant species richness to understand more specifically how relationships specific to plant growth patterns may shift under changing climate.

We extracted 27 individual intact plant–soil mesocosms using cores 30 cm in diameter and 40 cm in height (Berauer et al. 2019; Zistl‐Schlingmann et al. 2020). The intact mesocosms were extracted, randomized, transported, and reburied within bottomless plastic pipes. Pipes were used to limit soil physical properties to the original control conditions, delineate individual mesocosms, facilitate water movement, and allow development of deep roots. We incorporated a control that accounts for impacts of mesocosm removal by transplanting nine mesocosms within the origin site (Esterberg, 1260 m a. s. l.). Transplants from the origin site were inspected to ensure homogeneity of plant species and high species richness. As a result, mesocosms from the origin site comprised a median of 15 different plant species with a median Shannon evenness index of 0.75. Nine mesocosms were transplanted downslope to both Graswang (860 m a.s.l.) and Fendt (600 m a.s.l.). The total number of mesocosms was limited largely due to the difficulty and costs of extracting, transporting, and sampling from many mesocosms at multiple sites. Esterberg, Graswang and Fendt soils developed from calcareous material (Garcia‐Franco et al. 2020): Rendzic Phaeozem in Esterberg, Haplic Cambisol in Graswang and Fluvisol in Fendt (Table 1). All sites consisted of purely herbaceous vegetation in meadows with no tree canopy and less than 3 degrees of slope. The origin and translocation sites are all located > 50 m from the forest edge. Additional details regarding climatic, biotic, and edaphic variables for individual sites can be seen in Table 1.

**TABLE 1:** Description of the individual metrics of each category (plant, soil, nutrients) that were incorporated into our analysis. The unit describes the quantification of the metric.

Metric	Category	Unit
Total plant biomass	Plant	Abundance: grams dry biomass
Plant species evenness	Plant	Shannon index
Plant species richness	Plant	Count: unique species
Total plant N	Plant	Abundance: Percent N content multiplied by total mass
Community weighted mean of leaf C:N ratio	Plant	Ratio: total dry mass content of each element
Relative graminoid abundance	Plant	Ratio: total dry mass of graminoids relative to total dry mass
Relative forb abundance	Plant	Ratio: total dry mass of forbs relative to total dry mass
Relative legume abundance	Plant	Ratio: total dry mass of legumes relative to total dry mass
Ammonium	Soil Nutrient	Abundance: concentration
Plant available phosphorus	Soil Nutrient	Abundance: concentration
Soil pH	Soil Nutrient	Log concentration of Hydrogen ions
Total soil carbon: Total Soil N	Soil Nutrient	Ratio: total dry mass content
Soil organic carbon	Soil Nutrient	Abundance: concentration
Total soil N	Soil Nutrient	Abundance: concentration
Nitrate	Soil Nutrient	Abundance: concentration
Nitrite	Soil Nutrient	Abundance: concentration
Archaea	Soil Microbe	Abundance: copies of relevant gene
Ammonia oxidizing archaea	Soil Microbe	Abundance: copies of relevant gene
Bacteria	Soil Microbe	Abundance: copies of 16S_rRNA gene
Nitrogen mineralizers	Soil Microbe	Abundance: total copies of apr and chiA genes
Denitrifiers	Soil Microbe	Abundance: total copies of nirK, nirS, and nosZ genes
Nitrifiers	Soil Microbe	Abundance: copies of relevant gene for AOA and AOB
Fungi	Soil Microbe	Abundance: copies of IST gene
N Fixers	Soil Microbe	Abundance: copies of nifH gene

Transplants occurred at the beginning of the growing season during the first week of April 2016. Plant and soil data used in this analysis were collected across all mesocosms (*n* = 27) from one sample date on July 23–24, 2019, and thus represent plant and soil communities that have experienced nearly four growing seasons under novel climate. The origin site of the translocated mesocosms, used as a control, is a managed grassland that previously was cut once per year with summer grazing by cattle, but with no active slurry or mineral fertilizer application. Following translocations, we maintained the management schedule of one cutting per year by removing all vegetation greater than 3 cm (Ba et al. 2012; Andrade‐Linares et al. 2023). We excluded browsing by livestock from our mesocosms by use of an electric fence. All sites have similar topography and constitute a general classification as subalpine grasslands.

We selected eight individual metrics within each of the following three categories: plant communities, soil nutrient pools, and soil microbial communities (Table 2). Choice of metrics was made a priori and was guided by current understanding of taxa or metrics that are expected to play a role in plant–soil relationships. For plant metrics we included: total dry biomass, plant species richness, plant species evenness, total dry graminoid biomass, total dry legume biomass, total dry forb biomass, and community weighted means of leaf C:N and total plant nitrogen. For soil nutrient pool metrics, we included: ammonium, soil pH, C:N ratio, total N, soil organic C, plant available P, nitrate and nitrite. For soil microbial communities, we included: abundance of archaea, bacteria and fungi, as well as the abundance of microbes catalyzing key processes in the N cycle (mineralizers, nitrifiers, denitrifiers, and N fixers).

**TABLE 2:** Geographic, climatic, edaphic, and plant sociologic description of the three experimental sites, note we studied only communities originating from the two highest sites here. Climatic data is averaged across the years of the experiment (2016–2019).

	Experimental site		
	Fendt	Graswang	Esterberg
**Geographic characteristics**			
Elevational Belt	montane	montane	montane
Coordinates	47.82932° N 11.06626° E	47.56975° N 11.01434° E	47.51634° N 11.15773° E
Elevation [m a.s.l.]	600	860	1260
**Climatic characteristics**			
Air Temperature [°C]	9.1	7.1	6.3
Precipitation [mm‐year]	920	1424	1160
Nr of days of growing season*	240	227	219
**Edaphic characteristics**			
*Bulk density (g cm* ^*−3*^ *)*			
0–5 cm	0.6 ± 0.1	0.4 ± 0.1	0.5 ± 0.1
5–15 cm	0.9 ± 0.1	0.5 ± 0.1	0.6 ± 0.1
*EC (μS cm* ^*−3*^ *)*			
0–5 cm	405.4 ± 70.1	356.7 ± 103.7	367.6 ± 152.6
5–15 cm	251.3 ± 33.5	340.1 ± 68.9	304.5 ± 109.7
**Soil Type**	**Fluvisol**	**Haplic Cambisol**	**Rendzic Phaeozem**
*Texture [%]*			
Clay			
0–5 cm	37	60	62
5–15 cm	38	59	41
Silt			
0–5 cm	39	37	35
5–15 cm	35	38	51
Sand			
0–5 cm	24	3	3
5–15 cm	27	3	8
*CaCO*_*3*_ *(mg g* ^−*1*^ *)*			
5–15 cm	0.1 ± 0.0	13.2 ± 0.5	5.9 ± 3.2
0–5 cm	0.0 ± 0.0	11.6 ± 0.5	2.8 ± 1.6
*SOC (mg g* ^−*1*^ *)*			
0–5 cm	60.6 ± 7.8	133.9 ± 6.5	188.9 ± 6.2
5–15 cm	35.0 ± 2.8	116.2 ± 1.1	129.8 ± 12.4
*N* (mg g^−1^)			
0–5 cm	6.6 ± 0.5	12.4 ± 1.8	18.8 ± 2.7
5–15 cm	4.2 ± 0.4	10.0 ± 1.3	13.8 ± 2.9
*C:N‐ratio*			
0–5 cm	9.2 ± 0.7	10.8 ± 0.7	10.0 ± 1.2
5–15 cm	8.3 ± 0.6	11.6 ± 1.3	9.4 ± 1.4
**Plant association**	***Arrhenatherion elatioris***	***Trisetetum flavescens***	***Cynosurion cristati***
**Plant sociology**			
Dominant species	*Alopecurus pratensis*	*Dactylis glomerata*	*Anthoxanthum odoratum*
	*Dactylis glomerata*	*Festuca pratensis*	*Cynosurus cristatus*
	*Elymus repens*	*Festuca rubra*	*Elymus repens*
	*Lolium perenne*	*Trisetum flavescens*	*Festuca pratensis*
	*Poa angustifolia*	*Pimpinella major*	*Festuca rubra*
	*Poa pratensis*	*Plantago lanceolata*	*Lolium perenne*
	*Taraxacum officinalis*	*Trifolium pratense*	*Trifolium pratense*

* Growing season was defined as the period per year between first of 5 days with average temperature above 5°C and last harvest event of the year and was calculated for years 2017–2018. Soil type follows the definitions of (Food and Agriculture Organization of the United Nations 2014).

We approached our use of ammonium, nitrate, and nitrite concentrations with care, knowing that N pools can be ephemeral and transient. Though our samples only come from one point in time, we found their concentrations to be strong predictors of individual metrics of plant biomass and soil microbe abundance. These metrics explained more variance of microbial and plant metrics than the metric of total soil N. Because of the strong relationship, we retained these within our nutrient pool variables of interest for the subsequent analyses. To further investigate these trends, we provide a synthesis of previous findings within this experimental setup in the discussion to determine consistency among our results.

### 2.1 Climate Change Categories

Downslope transplants from our origin site to the two downslope recipient sites corresponded to warmer atmospheric conditions, periods of drier soil, and longer growing seasons (Berauer et al. 2021) (Table 2). The translocation from the origin site (Esterberg 1260 m a.s.l.) to the mid‐elevation site (Graswang 860 m a.s.l.) resulted in warmer conditions (+0.85°C) with years of similar or higher precipitation relative to the origin site (+20% average annual precipitation during the experiment) and longer periods with dry soil during a drought year (−24% mean soil moisture in 2018) (Berauer et al. 2021). Translocation from the origin site (Esterberg 1260 m a.s.l.) to the lowest site (Fendt 600 m a.s.l.) resulted in major warming across years (+2.9°C), with a decrease in mean annual precipitation during the experimental period (−20%) and longer periods with drier soils during a drought year (−33% mean soil moisture in 2018, estimated by Berauer et al. (2021)).

### 2.2 Plant Sampling and Processing

Plant biomass from each mesocosm was harvested by removing all plant biomass exceeding 3 cm in height, sorted to species, and then dried at 60°C for a minimum of 48 h to constant weight (Halbritter et al. 2019). This biomass represents annual plant productivity as each growing season started from the same point (3 cm) and the entire mesocosm was harvested at the end of each growing season. Plant species richness was based on the species identified in the sorted biomass.

To analyze leaf chemistry, we homogenized a representative mixture and quality of dried community aboveground biomass bulk samples by clipping away stems and reproductive tissue, shredding them to 2 mm (SK1, Retsch GmbH; Haan, Germany), and then pulverizing them with a ball mill (MM301, Retsch GmbH; Haan, Germany) (Halbritter et al. 2019). Carbon and N content (percentage of dry weight) of mixed community bulk samples was analyzed using elemental analysis (Thermo Quest Flash EA 1112, Thermo Fisher Scientific; Waltham, USA). Leaf P content (P in g kg ^−1^ equivalent to permille) was determined using inductively coupled plasma optical emission spectrometry (Vista‐Pro radial, Varian Inc.; Palo Alto, USA) after pressure digestion in 65% HNO_3_ to adhere to international standardized protocols (Bayreuth Center of Ecology and Environmental Research, central analytical chemistry laboratory; Bayreuth, Germany). We derived C:N, C:P, and N:P ratios (note, P was transformed to percent prior to calculation). Absolute amount metrics of each metric were calculated as the product of produced biomass times the relative portion of each respective element.

### 2.3 Soil Sampling

Soil samples were taken from all plant–soil monoliths located at each site by using disinfected stainless‐steel cores (5 cm diameter) to a depth of 7 cm. The top two‐cm layer consisting entirely of plant litter, was removed from each core. From each mesocosm two soil core samples (ca. 50 g soil) were homogenized by sieving through a 5 mm‐mesh sieve, and stones and roots were removed. A subsample of the homogenized soil was immediately collected in sterile falcon tubes (15 mL) and stored in dry ice for further DNA extraction and molecular analysis. The remnant soil was stored at 4°C for further microbial biomass and inorganic N analyses. Soil pH was measured in a 1:2.5 (soil: water) suspension of 10 g soil in 25 mL of 0.01 M CaCl_2_ solution.

### 2.4 Plant‐Available P, Organic C, and Total N Determination of Bulk Soil

Plant‐available P was extracted from sieved soil samples with 0.5 M NaHCO_3_ adjusted to pH 8.5 with NaOH (Olsen 1954). The total C (TC) and total N (tN) contents of bulk soils were determined using an Elemental Analyzer (Elementar, VarioMax cube, Langenselbold Germany). Inorganic C (IC) was determined in the same way after heating the samples in a muffle furnace (Carbolite, ELF 11/6B, Germany) at 550°C for 4 h to remove OC. Finally, the SOC content was calculated as the difference between the total C (TC) and the inorganic C (IC) content. The samples were analyzed in triplicates.

### 2.5 Microbial Biomass and Inorganic N Concentrations in Soil

Microbial biomass was measured by using a modified chloroform–fumigation extraction method (Vance et al. 1987) based on Brookes et al. (1985). In brief, 5 g of fresh soil was fumigated with chloroform vapor overnight. Subsequently, fumigated and nonfumigated samples were extracted with 20 mL of 0.01 M CaCl_2_ solution. To calculate microbial biomass C (C_mic_) and microbial biomass N (N_mic_), the total dissolved C and N concentrations of the nonfumigated extracts were subtracted from those of the fumigated samples and divided by the respective calibration factor (coefficient KE) for C_mic_ (KE_C_ = 0.45) and N_mic_ (KE_N_ = 0.54) (Joergensen and Mueller 1996). In addition, NH_4_^+^‐N, NO_3_^—^N, and NO_2_^−^‐N were measured from the nonfumigated extracts by a Continuous Flow Analyzer (Skalar 5100; Skalar Analytic, Germany).

### 2.6 DNA Extraction

DNA was extracted from 0.5 g fresh weight of soil samples following a modified phenol‐chloroform extraction procedure (Töwe et al. 2011) with the Precellys24 Instrument (Bertin Technologies, France). The extraction quality of all the samples (final volume 50 μL) was estimated by absorbance ratio calculations (A260/A280 and A260/A230) using a UV–Vis spectrophotometer (NanoDrop ND‐1000; Thermo Fischer Scientific, USA). DNA concentrations were determined using the Qubit 4 fluorometer after dyeing selectively double‐stranded DNA with the Qubit dsDNA BR Assay kit following the instructions of the manufacturers (Life Technologies, USA). The DNA concentrations were in the range of 148–1640 ng μL^−1^. DNA samples were stored at −80°C until further processing.

### 2.7 Quantitative Real‐Time PCR Assay

Marker genes for the microbial community involved in the mineralization of N (*apr* and *chiA* genes), the oxidation of ammonia (*amoA* gene for archaea [AOA] and bacteria [AOB]), denitrification (*nirK*, *nirS*, and *nosZ* genes), and N_2_ fixation (*nifH* gene) were quantified by quantitative Real‐Time PCR (qPCR). In addition, the 16S‐rRNA gene for bacteria and archaea as well as the ITS region for fungi were included to quantify those groups. Separated qPCRs were carried out using SYBR green as fluorescent dye and performed on a 7300 Real‐Time PCR System (Applied Biosystems, Germany). A pre‐experiment was first conducted to avoid PCR inhibition due to the soil sample. This resulted in an optimal sample dilution of 1:256 for all the samples (data not shown). Details on the marker genes and qPCR conditions are described in Andrade‐Linares et al. (2021). Serial plasmid dilutions (10^1^–10^7^ gene copies mL^−1^) were used for standard curve calculations. The PCR efficiency percentages of the amplifications (Efa %) were calculated from the standard curve by the formula Efa % = [10 (−1/slope) – 1] × 100% (Töwe et al. 2011). Efa % resulted respectively for each marker gene in the following values: for alkaline proteases (*apr*) 81%, chitinases (*chiA*) 84%, *amoA* AOA 84% and AOB 82%, *nirK* 91%, *nirS* 83%, and *nos*Z 92%, and *nifH* 88%. The qPCR efficiencies for quantification of bacteria, archaea, and fungi were 87%, 81% and 82%, respectively. The coefficient of determination (*R* ^2^) of the standard curves was determined to be above 0.99 for each qPCR. The specificity of the amplified products was checked by melting curves of the amplicons and 2% agarose gels. To account for a missing measurement of AOB abundance in one mesocosm, we estimated the value using a linear regression (*R* ^2^ = 0.38) with fixed factors of total bacteria abundance, translocation treatment, and AOA. This approach enabled the inclusion of all measurements across all mesocosms in the multivariate analysis.

### 2.8 Statistical Models

To compare the strength of relationships between plant community metrics, soil nutrient pools, and soil microbial communities, we used Mantel correlations between variable sets in control, moderate climate change, and major climate change translocation treatments. All analyses were performed in R (R Core Team 2024). Distance matrices for the Mantel correlations were calculated as the Bray–Curtis dissimilarity metric for the plant microbial community metrics, and Euclidean distance for the nutrient pools. Our choice of dissimilarity and distance metric (Bray–Curtis vs. Euclidean) was made according to which metric explained more variance within each matrix. The Mantel correlations identify if changes in one group of metrics (plants, microbes, soil nutrients) are correlated with changes in another group of metrics. Correlation metrics and their statistical significance are determined by iterating 1000 permutations. Only one of the two distance matrices is permuted while the other is held constant.

To identify the strength and direction of relationships of individual soil microbial functional groups and specific soil nutrients on annual plant productivity and plant species richness, we used a Spearman correlation metric. For this specific analysis, our study used a significant *p* value threshold of 0.1 following a power test using simulated data differences with the sample size and expected variance from our project that was unable to statistically detect differences with an alpha level of 0.05.

To test for significant changes in the abundances of individual metrics from the control group, we used an ANOVA with a pairwise comparison of control versus moderate and major climatic change groups. For display purposes only, we show the values of downslope translocation treatments relative to the mean of the control group. The statistical results are derived from the original pairwise comparisons that compare the original abundance metrics of each translocation treatment against the control group. All model results from correlations and the ANOVA used Tukey HSD *p* value correction to account for potential error bias when conducting multiple tests/comparisons.

## 3 Results

We observed substantial changes in individual metrics of the plant community, soil nutrient pools, and the abundance of various microbial groups in the soil, relative to mesocosms under control conditions (Figures 3, 4, 5). Across translocation treatments, we also observed shifts in the strength and significance of Mantel correlations between plant community metrics, nutrient pools, and the abundance of microbial functional groups, indicating that environmental changes significantly altered plant–soil relationships (Figure 4).

### 3.1 General Relationships Between Plants and Soil

**FIGURE 1:**
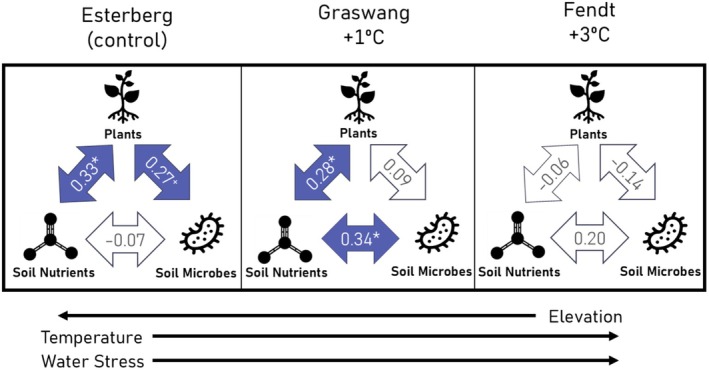
Mantel correlations between multivariate datasets of plant community characteristics, soil nutrient pools, and microbial community characteristics at the control site and two downslope translocation sites. Significant (*p* < 0.05, indicated by asterisk) or near significant (*p* < 0.1, indicated by plus symbol) correlations between distance matrices across mesocosms are blue, while insignificant correlations are white. Positive values indicate the differences in one group of metrics are related to a change in another. Negative values indicate that changes in one group of metrics are related to homogeneity of the other.

Under control conditions, changes in plant community metrics among mesocosms were related to changes in both soil microbial community abundance and soil nutrient pools (Figure 1). Following moderate environmental change (+1°C, +8 days to growing season), shifts in plant community metrics among mesocosms remained linked to soil nutrient pools, although the direct relationship with the soil microbial community diminished (Figure 1). Under major environmental change (+3°C, +21 days to growing season), changes in plant community metrics among mesocosms were no longer significantly related to shifts among soil nutrient pools or soil microbial community (Figure 1). In fact, major climate change led to a sign change (statistically insignificant) in the Mantel correlations between plant community and soil nutrients. This suggests that increased dissimilarity in one group (e.g., plants, soil nutrient pools, microbes) was more often associated with more homogeneous (similar) conditions in another group.

### 3.2 Specific Relationships Between Soil and Plant Metrics of Productivity and Richness

**FIGURE 2:**
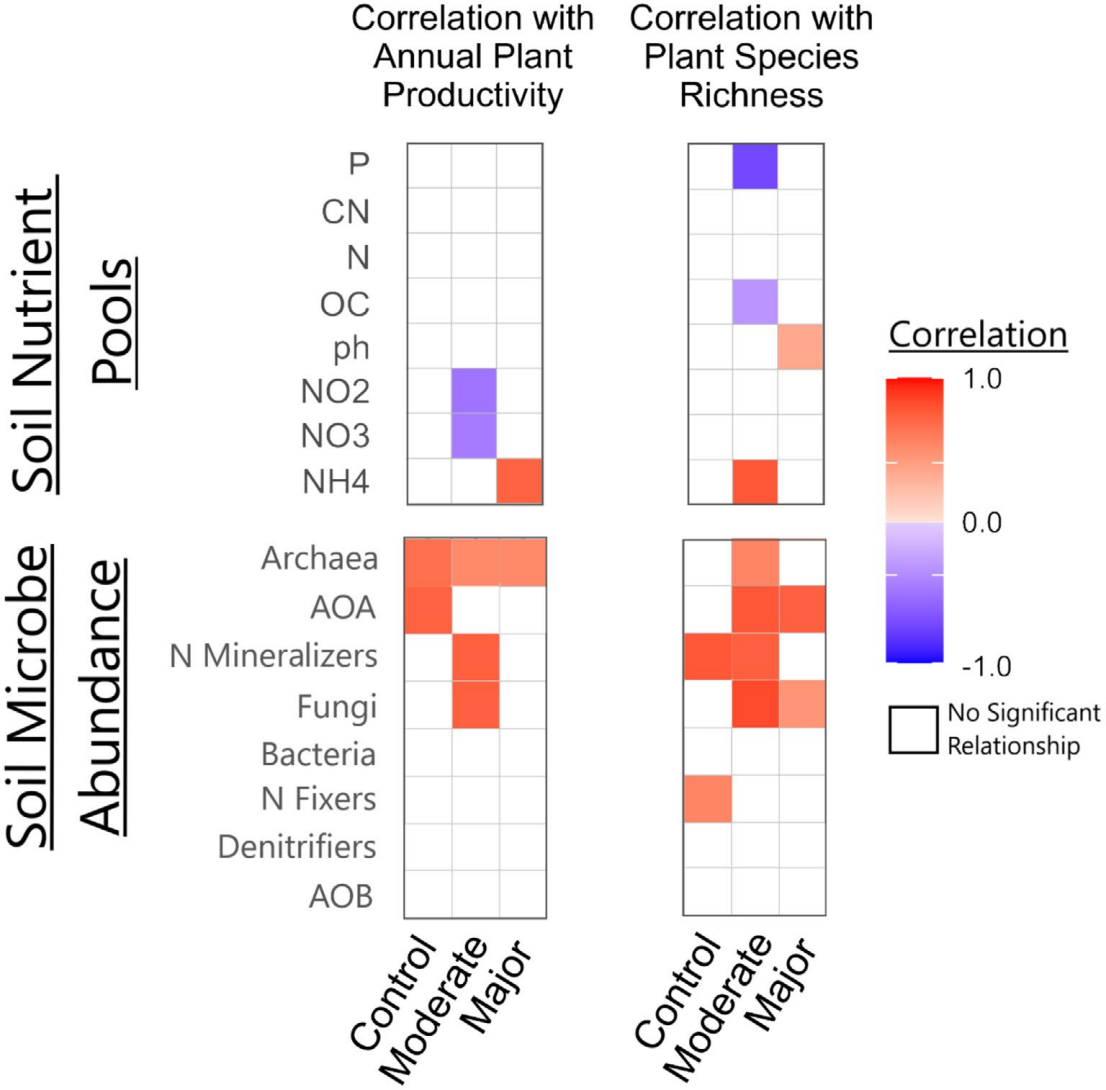
Correlation values between individual metrics and annual plant productivity (left) and plant species richness (right). Individual columns indicate the identity of the translocation. Colors indicate the strength and direction of the correlation within each site/translocation. White spaces indicate no significant (*p* < 0.1) correlation. AOA, ammonium oxidizing archaea; AOB, ammonium oxidizing bacteria; OC organic carbon.

We found strong shifts in multivariate relationships (Figure 1), we also found that plant productivity was significantly correlated with multiple microbial groups and nutrient pools. However, the strength and significance of the correlation values varied largely following moderate and major climate change. Notably, archaea biomass consistently showed a significant correlation with total plant biomass across all conditions (Figure 2). Fungi and N mineralizer biomass were also key factors positively correlated to annual plant productivity under moderate change, but these relationships weakened under major environmental change (Figure 2). In contrast, nitrate and nitrite concentrations became negatively correlated with annual plant productivity under moderate climate change (Figure 2). Among the nutrient pools, ammonium concentration was the only strong factor related positively to annual plant productivity under major environmental change (Figure 2).

Plant species richness was also correlated with multiple microbial groups and nutrient pools; however, these relationships differed from those observed with plant productivity. Under control conditions, all nutrient pools exhibited positive but statistically insignificant correlations with plant species richness. However, N mineralizers and N fixers were positively correlated with plant species richness under control conditions (Figure 2). Following moderate climate change, plant species richness was significantly negatively correlated with total plant‐available P, soil organic C, and significantly positively correlated with ammonium concentrations (Figure 2). Following major climate change, plant species richness was highly correlated with the abundance of archaea, AOA, N mineralizers, and fungi in soil (Figure 2).

### 3.3 Plant Community Metrics

**FIGURE 3:**
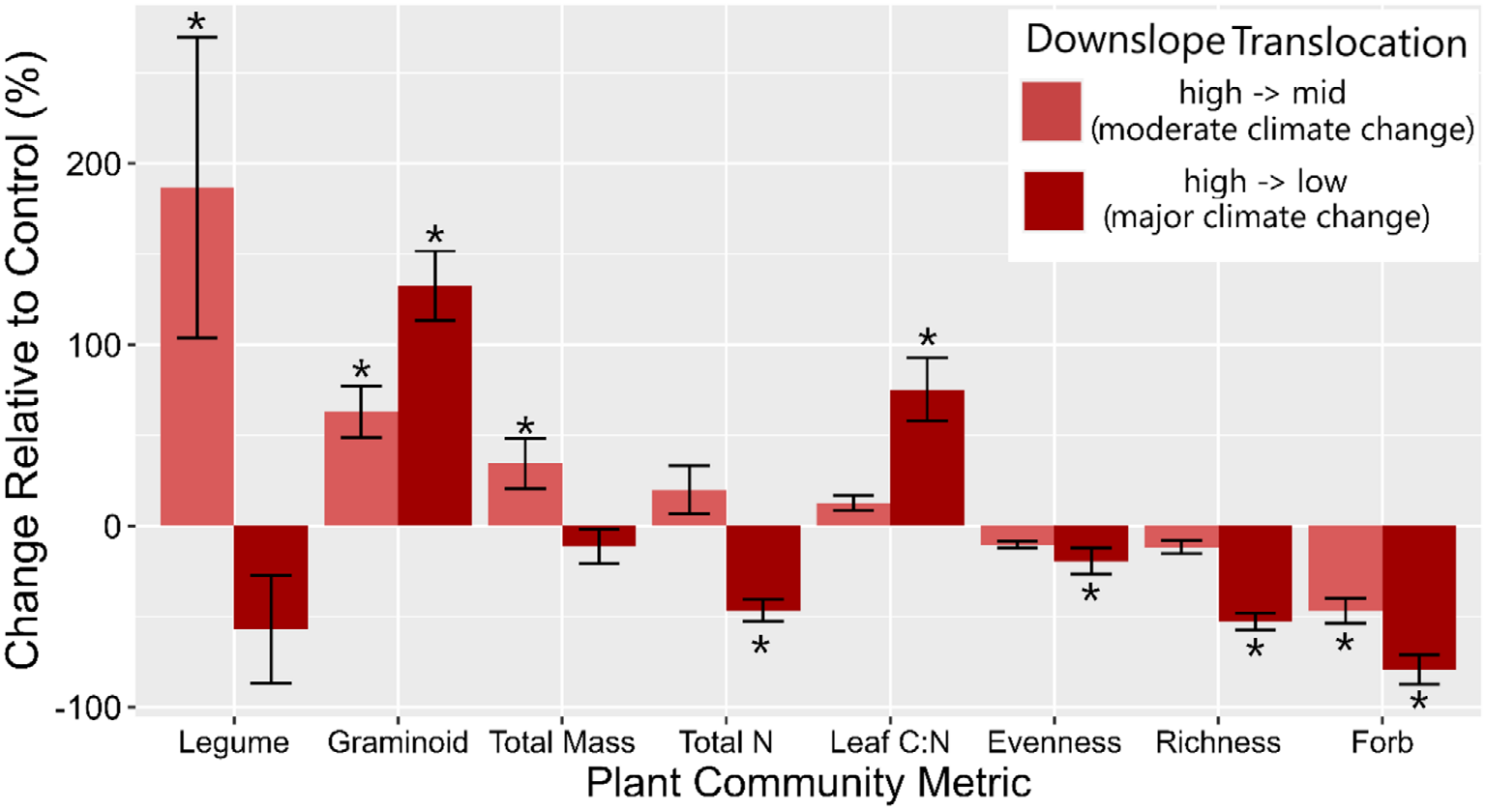
Response of plant community metrics to downslope translocations resulting in moderate and major climate change. Values are relative to the control group (same community sampling but not translocated downslope) 4 years following translocation. Significant differences from the control group are indicated by an asterisk.

Among the various plant community metrics, most exhibited positive responses to moderate climate change (translocation from high to mid elevation, +1°C), with significant increases in annual plant productivity (38%), relative graminoid abundance (64%), and relative legume abundance (188%) (Figure 3). Forb abundance was the only plant metric to significantly decrease (−48%) under moderate warming. Following major climate change (translocation from high to low elevation, +3°C) plant species richness (−53%), species evenness (−16%), forb abundance (−69%), and total plant N (−49%) significantly declined compared with the control, while relative graminoid cover (+106%) and leaf C:N ratios (+72%) increased (Figure 3).

### 3.4 Soil Nutrient Pools

**FIGURE 4:**
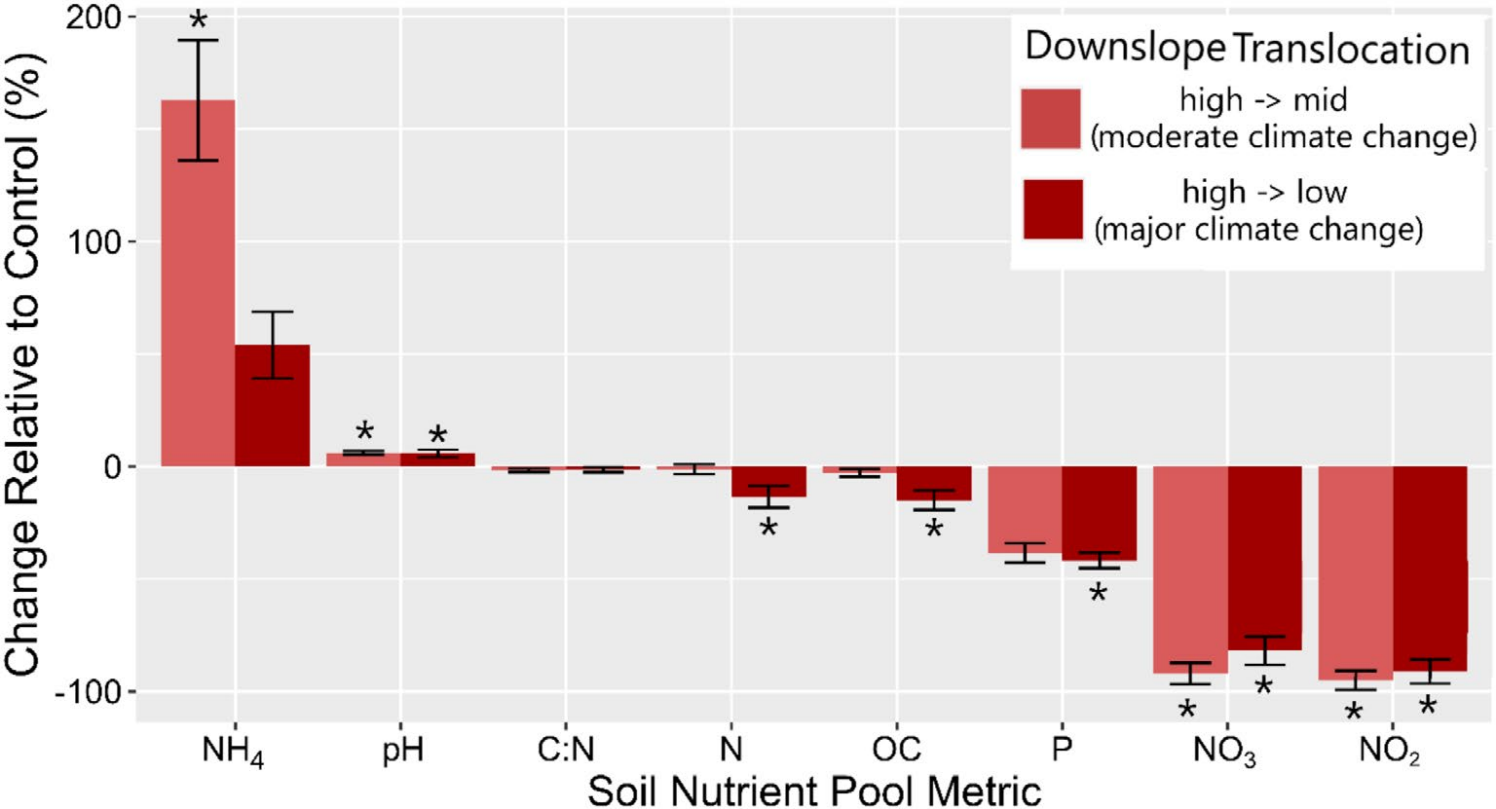
Response of soil nutrient pools to downslope translocations resulting in moderate and major climate change. Values are relative to the control group (same community sampling but not translocated downslope) 4 years following translocation. Significant differences from the control group are indicated by an asterisk.

Moderate and major climate change had comparable effects on soil nutrient pools, with major warming amplifying the declines observed under moderate warming, except for ammonium concentrations, which did not follow this trend (Figure 4). Following moderate warming, we observed significant increases in ammonium concentrations (+165%) and soil pH (+8%) and significant decreases in nitrate (−90%) and nitrite (−93%) concentrations (Figure 2). Major climate change reduced the total soil N (−16%), organic carbon (−17%), plant available phosphorus (−39%), nitrate (−78%), and nitrite (−80%) and increased soil pH (+8%) compared with the control group (Figure 4).

### 3.5 Soil Microbial Community

**FIGURE 5:**
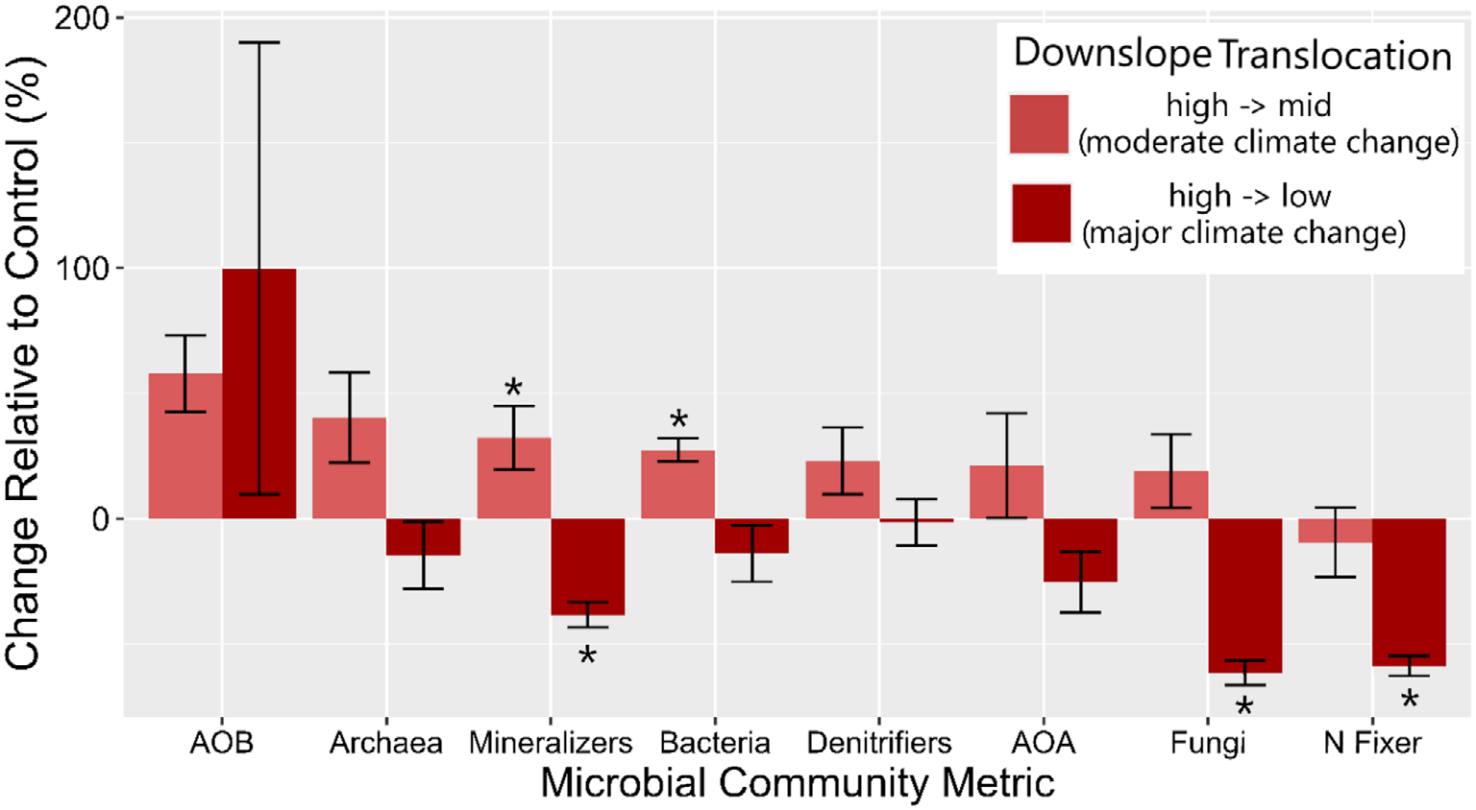
Response of microbial community metrics to downslope translocations resulting in moderate and major climate change. Values are relative to the control group (same community sampling but not translocated downslope) 4 years following translocation. Significant differences from the control group are indicated by an asterisk.

The abundance of most soil microbial groups differed considerably between moderate and major climate change effects. The biomass of all soil microbes in our analysis (apart from N fixers) generally increased under moderate environmental change, although the increases were not always statistically significant (Figure 5). In contrast, following major climate change, total microbial biomass declined across most groups with significant reductions observed in N mineralizers (−36%), N fixers (−57%), and fungi (−62%) compared to the control (Figure 5).

## 4 Discussion

Our study documents significant shifts in plant community metrics, soil nutrient pools, and microbial communities following downslope translocation of intact plant–soil mesocosms to warmer and drier conditions. We found that the multivariate relationships between plant metrics, soil pools, and microbes shifted under climate change scenarios. Regarding our first hypothesis: The strength of plant‐microbe‐soil nutrient interactions will increase under both moderate and major environmental changes, as plants increasingly depend on microbially mediated nutrient cycling under altered abiotic conditions. We observed slight changes in direct relationships under moderate climate change; however, major climatic changes resulted in a pronounced dissolution of both direct and indirect relationships (Figure 1). Our second hypothesis posited that microbial plant–soil relationships will become increasingly critical for maintaining plant productivity and species richness in response to environmental change. We expected that specific plant–soil relationships that are important for plant productivity and species coexistence under control conditions will become more important following environmental change. Again, we observed that many strong positive plant–soil relationships emerged under moderate change, but largely dissipated under major changes, suggesting threshold‐like responses (Figure 2).

### 4.1 Plant–Microbe Linkages Under Environmental Change

Under control conditions, plant‐microbe relationships were significant, with microbes and plants influencing each other directly. Under moderate change conditions, these relationships became indirectly important, through microbial effects on soil nutrient pools. However, under major environmental change, plant–microbe linkages were largely dissociated, as indicated by Mantel correlations (Figure 1). This suggests that plants may increasingly decouple from microbial communities beyond certain climatic thresholds. Additionally, under moderate and major change, we observed both significant and insignificant increases in annual plant productivity, accompanied by the reduction in the direct influence of microbes on plant biomass (Figures 2 and 3). This decoupling could signal a shift in plant–soil feedback (PSF) mechanisms. Under moderate change, our correlation analysis (Figure 2) also shows that strong beneficial relationships between individual soil microbial groups and plant growth were most prevalent, but a shift in sign (from negative to positive) under major change was not observed, indicating that these relationships might become more neutral. These more neutral relationships may be due to many potential reasons including a more balanced distribution of pathogenic and beneficial taxa within general microbial groups like bacteria, decreases in microbial activity/diversity rather than abundance, or the loss of specific plant or microbial taxa that previously drove the positive trend.

Under moderate climate change, fungi emerged as strong positive contributors to plant productivity. However, the influence of fungi diminished under major climatic changes, likely due to a combination of thermal stress and drought conditions. Fungi are thought to improve water and N acquisition for plants in warmer climates but are also sensitive to drought (de Vries et al. 2023). Overall fungi abundance and its relationship with plant growth decreased under major environmental change (Figures 1 and 5), a translocation that included both warmer temperatures and exposure to more intense drought conditions during one of the four growing seasons (Table 2). However, we are unable to determine whether the change in fungi abundance and its relationship to plant growth is due to the effect of temperature, periods of dry soils, or both.

Fungi and N mineralizers may exhibit optimal temperature ranges for activity, with moderate warming (+1°C) enhancing their metabolic rates, resulting in more efficient decomposition of soil organic matter and nutrient release, aligning with the observed increases in plant productivity. However, major warming (+3°C) could push these organisms beyond their thermal threshold, causing thermal stress. This may reduce their efficiency or activity and potentially shift community composition toward less efficient decomposers, thereby reducing their impact on grassland productivity. Additionally, it is possible that despite decreases in microbial biomass of key groups, microbial activity of the microbes might still increase under warmer conditions (Gao and Yan 2019), but our study does not directly measure microbial activity, only abundance of relevant genes.

Under moderate and major environmental change, we found a consistent positive correlation between plant productivity and archaea. Archaea could be particularly beneficial under warmer soil conditions due to their tolerance of higher temperatures, abiotic stress, and the direct influence on N, C, S, and P cycling (Jung et al. 2020). Their resilience and activity under abiotic stress may underpin that their importance in maintaining plant community productivity under warmer conditions is likely due to the role of archaea in N cycling. Archaea's adaptability to higher temperatures, and their ability to maintain or increase nutrient availability under stressful conditions has been shown to support the productivity of plants in a warmer climate (Huang et al. 2021). However, the role of archaea in grassland plant growth is still not completely understood.

### 4.2 Plant–Nutrient Linkages Under Environmental Change

Nutrient pools were important determinants of plant productivity and species richness in both control and moderate climate change scenarios but became less important under major environmental change (Figure 4). This may reflect the loss of plant community diversity, particularly forbs, along with the decrease in plant species richness and evenness (Figure 3). Leaf C:N ratios remained consistent across control conditions and moderate climate change (Figure 3), but soil nutrient pools did not (Figures 3 and 4).

Under moderate and major environmental change, soil nitrate concentrations declined drastically (Figure 4), potentially by increased plant N uptake due to an increase in graminoid dominance and higher N_2_O emissions (Dai et al. 2020; Hu et al. 2024). Additionally, we observed negative correlations of annual plant productivity with soil nitrate and nitrite concentrations following downslope translocation (Figure 2). We hypothesize that this may indicate that plant uptake of nitrate might have either (1) increased while nitrification rates remained stable or dropped or (2) remained stable while nitrification rates decreased under climate change. We find the first of these hypotheses to be more likely given that annual plant productivity increased, especially under moderate climate change where biomass of nitrifiers did not. Moreover, the observed growth shifts were largely driven by increased growth of graminoids that exhibit acquisitive growth strategies and respond favorably to N availability (Figure 3). Consistent with our findings, previous studies have shown that when warming increases plant growth, it can lead to nutrient mining, if fertilizer or organic matter inputs do not replace the soil nutrients removed by plants (Wang et al. 2016; Zistl‐Schlingmann et al. 2020). However, our results constitute a one‐time sampling of ammonium, nitrate, and nitrite concentrations providing a small snapshot of these often‐variable nutrient pools. These results, while linked with plant and microbe function, should be further investigated to better understand the temporal dynamics of nitrogen in its various forms following climatic change.

Soil ammonium (NH_4_^+^) concentrations did not decrease like nitrate and nitrite but rather increased under moderate change (Figure 2), mirroring previous studies that show N mineralization rates may increase under warmer soils (Dai et al. 2020; Andrade‐Linares et al. 2021). This may be due to a shift toward a more optimal growth temperature that increases plant N uptake (Jayawardena et al. 2021). Ammonium uptake may have become less favorable under high temperatures in these grasslands, aligning with some evidence for disproportional increases in nitrate versus ammonium absorption (Bassirirad 2000; Hu et al. 2024). Nitrate is often the preferred version of N for uptake as it is free to move within the root solution due to the tendency for soils to possess an overall negative charge (Miller and Cramer 2005). Ammonium uptake is thought to be preferred at lower temperatures and nitrates at higher temperature (Bassirirad 2000), potentially shifting plant N acquisition strategies to use more nitrates than ammonium.

### 4.3 Microbe–Nutrient Linkages Under Environmental Change

Nutrient pools were more strongly correlated with microbial abundances under moderate change than under control or major climate change scenarios (Figure 1). Although our study focused solely on nutrient pools, it is well documented that nutrient fluxes, including processes such as mineralization, respiration, denitrification, and nitrogen fixation, can increase or decrease under warmer conditions (Dai et al. 2020; Hu et al. 2024). This can be due to higher microbial metabolism, greater C inputs, and shifts from anabolic to catabolic processes (Dai et al. 2020). As soil temperatures rise, microbial activity generally increases, which can lead to higher rates of organic matter decomposition (Risch et al. 2019; Xu et al. 2023). This process releases ammonium as an intermediate product during the mineralization of organic N, particularly in conditions where soil moisture is limiting (Zhang et al. 2024). This may, in part, explain our results of high accumulation of ammonium in the soil under moderate warming (Figure 4).

Warming may also reduce the rate of nitrification, resulting in lower nitrate and nitrite levels, but studies do not indicate a clear pattern between nitrification and warming (Barnard et al. 2005) or periods of dry soils (Hartmann et al. 2013; Fuchslueger et al. 2014). Conversely, higher temperatures might also enhance denitrification (the process by which nitrate and nitrite are converted to nitrogen gases like N_2_ and N_2_O), particularly under anaerobic conditions, to decrease the concentrations of nitrate and nitrite in the soil. However, links between denitrification and warming have, in some cases, also been shown to be insignificant (Barnard et al. 2005). Downslope translocation also increased exposure to drought conditions that translated into longer periods of dry soil during one of the four experimental years. These dry conditions, combined with a rewetting, can enhance denitrification in managed grasslands (Harris et al. 2021), potentially explaining the decline in nitrate pools. Under major environmental change we observed not only significant reductions in nitrate (−78%), and nitrite (−80%), but also in total soil N (−14%), soil organic C (−16%), and soil P (−41%), while soil pH increased (+5%) (Figure 4). All these metrics are directly or indirectly related to an accelerated organic matter decomposition rate (Garcia‐Franco et al. 2024).

### 4.4 Asymmetric Responses to Moderate and Major Environmental Change

The relationships between groups (plants, microbes, soil nutrients) often differed in strength under moderate versus major climatic changes, which highlights potential thresholds in ecosystem responses (Figures 1 and 2). This may be due to changes in resource demands across multiple trophic levels that may have caused a mismatch of services and/or competition. We observed that the larger temperature increase resulting from translocation from high elevation to low elevation was not accompanied by additional precipitation, leading to both warmer and drier conditions (Table 2, (Berauer et al. 2021)). This combination is thought to result in different PSF effects compared to warming alone (van der Putten et al. 2016). Our largest downslope translocation (major environmental change) also resulted in a more stressful environment characterized by higher temperatures and longer periods of dry soil (Dai et al. 2020; Hu et al. 2024).

These results highlight potential limitations to generalizing relationships across environmental gradients, such as those predicted by the stress‐gradient hypothesis, which postulates that environmental shifts toward stressful conditions also shift biotic relationships from competitive to facilitative (Maestre et al. 2009). Though the stress‐gradient hypothesis has previously focused largely on plant–plant interactions, evidence of its application to plant–microbe interactions is emerging (David et al. 2020; Hernandez et al. 2021). Our study supports this idea but only following moderate climate change where we observed an increase in positive plant‐microbe relationships (Figure 2). Our results also partially align with predictions (van der Putten et al. 2016) that PSF can become more positive when novel environmental conditions enhance decomposition rates. While this pattern was evident under moderate environmental change, it was not observed following major climate change where relationships diminished.

### 4.5 Comparing Results to Previous Studies

Although our approach lacks the temporal depth of data needed to track long‐term plant–soil relationships, our findings regarding soil nutrient pools, microbial communities, and plant communities are validated in preceding and subsequent sampling efforts utilizing the same experimental design, at the same sites, but with differing levels of land management (fertilizer and mowing). Under low management intensity, these studies show an adaptive or limited response of biotic and abiotic components to moderate climate change, and a significant or large response following major climate change (Schuchardt et al. 2021; Andrade‐Linares et al. 2021, 2023; Zhou et al. 2024). Namely, they observed climate‐induced declines after two growing seasons in total phosphorus, total dissolved nitrogen, and dissolved organic carbon only following major climatic change. Moreover, the declines in total soil nitrogen observed in our study were also indicated in sampling of the subsequent year of our study (Garcia‐Franco et al. 2024). Plant communities exhibited similar shifts in growth, with graminoid dominance driving increases in productivity at moderate climate change and no changes or declines with major climatic change after 1–3 years (Berauer et al. 2019; Schuchardt et al. 2023).

Belowground indicators showed critical stress under major climate change after three growing seasons; microbial specific growth rates slowed by 25% (a shift toward K‐strategists), and resource limitation abruptly shifted from N‐limitation to Carbon (C) and Phosphorus (P) co‐limitation (Zhou et al. 2024). Major climate change was accompanied by a significant drop in Arbuscular Mycorrhizal Fungi (AMF) and P‐ mineralizer abundance (Andrade‐Linares et al. 2023). Collectively, these results illustrate an ecological collapse under severe warming and drying, but not under moderate climatic change. The unique difference seen from a previous study includes an increase in N‐mineralizer abundance following major climate change in the first year of the experiment (Andrade‐Linares et al. 2021). This effect was likely short‐lived, as our results indicate that N‐mineralizers declined under major climate change and increased under moderate climate change. Moreover, subsequent sampling indicating a decline in total nitrogen in another study (Andrade‐Linares et al. 2023) as well as our results (4 growing seasons after treatment).

## 5 Conclusions

This study underscores the potential for threshold‐like responses of natural systems to environmental change, specifically indicating that plant–soil relationships may shift under moderate climate change but weaken significantly under major climate change. While we did not observe a collapse in plant productivity, there was an intensified negative response of plant species' richness and evenness following major environmental change that cooccurred with the weakening of relationships between plant communities, soil nutrient pools, and microbial communities (Figures 1 and 2). These findings align with IPCC projections, which warn of severe climate impacts when warming exceeds 1.5°C. Our results should be viewed as an initial data point as our approach lacks frequent temporal sampling to document how individual variables and plant–soil relationships may diminish over time. We advocate for more studies that examine multiple levels of environmental change to better understand the resilience of relationships, rather than focusing solely on individual metrics or biotic groups. Moreover, our results indicate that beneficial and/or antagonistic plant–soil relationships may diminish under extreme climate shifts associated with warmer and drier conditions, which might further erode C stability. We encourage further investigation to explore how these plant–soil relationships shift under novel climate, with particular attention needed across large climatic gradients.

## Author Contributions

**Tyson J. Terry:** conceptualization (lead), formal analysis (lead), writing – original draft (lead), writing – review and editing (lead). **Peter Wilfahrt:** conceptualization (equal), investigation (equal), methodology (equal), writing – review and editing (equal). **Yujie Niu:** investigation (equal), methodology (equal), writing – review and editing (equal). **Diana R. Andrade‐Linares:** conceptualization (equal), methodology (equal), writing – review and editing (equal). **Khatab Abdalla:** conceptualization (equal), visualization (equal), writing – review and editing (equal). **Bernd J. Berauer:** conceptualization (equal), formal analysis (equal), project administration (equal), writing – review and editing (equal). **Michael Dannenmann:** conceptualization (equal), funding acquisition (equal), project administration (equal), writing – review and editing (equal). **Noelia Garcia‐Franco:** funding acquisition (equal), investigation (equal), methodology (equal), project administration (equal), writing – review and editing (equal). **Jincheng Han:** investigation (equal), writing – review and editing (equal). **Andreas von Hessberg:** funding acquisition (equal), investigation (equal), methodology (equal), project administration (equal), supervision (equal). **Elisabeth Ramm:** investigation (equal), methodology (equal), writing – review and editing (equal). **Ralf Kiese:** conceptualization (equal), funding acquisition (equal), investigation (equal), methodology (equal), supervision (equal), writing – review and editing (equal). **Ingrid Kögel‐Knabner:** conceptualization (equal), funding acquisition (equal), methodology (equal), supervision (equal), writing – review and editing (equal). **Michael Schloter:** funding acquisition (equal), investigation (equal), methodology (equal), project administration (equal), writing – review and editing (equal). **Martin Wiesmeier:** conceptualization (equal), funding acquisition (equal), methodology (equal), project administration (equal), supervision (equal), writing – review and editing (equal). **Stefanie Schulz:** investigation (equal), methodology (equal), project administration (equal), writing – review and editing (equal). **Anke Jentsch:** investigation (equal), methodology (equal), writing – review and editing (equal).

## Funding

This work was supported by the Federal Ministry of Education and Research (BMBF) BonaRes, 031B0027C, 031B0516C, 031B1067C.

## Conflicts of Interest

The authors declare no conflicts of interest.

## Data Availability

The data that support the findings of this study are openly available in the Open Science Framework at https://osf.io/6zjbn/.
