# Correction to “Plant–Soil Relationships Diminish Under Major Versus Moderate Climate Change in Subalpine Grasslands”

**DOI:** 10.1002/ece3.73164

**Published:** 2026-02-23

Terry, T. J., P. Wilfahrt, D. R. Andrade‐Linares, et al. 2025. “Plant–Soil Relationships Diminish Under Major Versus Moderate Climate Change in Subalpine Grasslands.” *Ecology and Evolution* 15, no. 12: e72578. https://doi.org/10.1002/ece3.72578.

Diana R. Andrade Linares, Michael Schloter, and Stefanie Schultz had an incorrect affiliation “Environmental Microbiology Research Group‐ EMRG, Biological Sciences Department, University of Limerick, Limerick, Ireland.”

The correct affiliation is “Comparative Microbiome Analysis; Helmholtz Munich, Ingolstaedter Landstr. 1, 85754 Oberschleissheim, Germany.”

We apologize for this error.

